# Preclinical Validation of SilkBridge^TM^ for Peripheral Nerve Regeneration

**DOI:** 10.3389/fbioe.2020.00835

**Published:** 2020-08-07

**Authors:** Federica Fregnan, Luisa Muratori, Giulia A. Bassani, Alessandro Crosio, Marco Biagiotti, Valentina Vincoli, Giacomo Carta, Pasquale Pierimarchi, Stefano Geuna, Antonio Alessandrino, Giuliano Freddi, Giulia Ronchi

**Affiliations:** ^1^Department of Clinical and Biological Sciences, University of Turin, Turin, Italy; ^2^Neuroscience Institute Cavalieri Ottolenghi, University of Turin, Turin, Italy; ^3^Silk Biomaterials Srl, Lomazzo, Italy; ^4^Department of Orthopaedics and Traumatology for Hand, ASST Gaetano Pini, Milan, Italy; ^5^Institute of Translational Pharmacology, National Research Council, Rome, Italy

**Keywords:** silk fibroin, nerve conduit, mechanical properties, *in vivo*, long-term biocompatibility, biodegradation, nerve regeneration, functional recovery

## Abstract

Silk fibroin (*Bombyx mori*) was used to manufacture a nerve conduit (SilkBridge^TM^) characterized by a novel 3D architecture. The wall of the conduit consists of two electrospun layers (inner and outer) and one textile layer (middle), perfectly integrated at the structural and functional level. The manufacturing technology conferred high compression strength on the device, thus meeting clinical requirements for physiological and pathological compressive stresses. As demonstrated in a previous work, the silk material has proven to be able to provide a valid substrate for cells to grow on, differentiate and start the fundamental cellular regenerative activities *in vitro* and, *in vivo*, at the short time point of 2 weeks, to allow the starting of regenerative processes in terms of good integration with the surrounding tissues and colonization of the wall layers and of the lumen with several cell types. In the present study, a 10 mm long gap in the median nerve was repaired with 12 mm SilkBridge^TM^ conduit and evaluated at middle (4 weeks) and at longer time points (12 and 24 weeks). The SilkBridge^TM^ conduit led to a very good functional and morphological recovery of the median nerve, similar to that observed with the reference autograft nerve reconstruction procedure. Taken together, all these results demonstrated that SilkBridge^TM^ has an optimized balance of biomechanical and biological properties, which allowed proceeding with a first-in-human clinical study aimed at evaluating safety and effectiveness of using the device for the reconstruction of digital nerve defects in humans.

## Introduction

Peripheral nerves are widely spread throughout the body and are therefore highly vulnerable to injury as a consequence of multiple causes, i.e., car accidents, domestic falls, military and sports injuries. Iatrogenic injuries and injury associated with degenerative conditions or diabetes are also very frequent (Dahlin et al., 2008a; Dahlin et al., 2008b; Cederlund et al., 2009; Haastert-Talini and Dahlin, 2018).

Peripheral nerve injuries affect 2,8% of trauma patients, many of which acquire lifelong disability (Noble et al., 1998; Taylor et al., 2008; Miranda and Torres, 2016). More than 300.000 peripheral nerve injuries are reported each year in Europe and over one million worldwide (Daly et al., 2012) and represent a major cause for morbidity, bringing to total or partial loss of motor, sensory and autonomic functions, with a devastating impact on a patients’ quality of life, especially for severe nerve injury. Associated healthcare costs are higher than €2,2 billion/year (Ciardelli and Chiono, 2006), and include not only the treatment, but also care and rehabilitation.

Although the peripheral nervous system has an intrinsic capacity to regenerate, this ability is often not sufficient and microsurgical intervention is therefore required. For short gap injures (<5 mm), a direct end-to-end tension-free repair between the two nerve ends is usually the chosen treatment. For longer gaps (>5 mm), a graft must be used to bridge the gap between the two nerve stumps and to guide regenerating axons towards target organs. Autologous nerve graft still represents the “gold standard” technique for bridging nerve defects and provides the best properties for best achievable functional restoration (Siemionow and Brzezicki, 2009; Kornfeld et al., 2019). However, it is associated with some drawbacks, including donor nerve morbidity, the need of an additional surgery to harvest the donor nerve that may be harmful to the patients, mismatch of donor nerve size with recipient site due to structural differences and limited availability of graft material (Ray and Mackinnon, 2010; Kornfeld et al., 2019).

Is therefore necessary to develop new strategies to find a suitable alternative to autologous nerve graft. In the last decades, research has focused on developing artificial nerve guide conduits (NGCs) in terms of materials selection and design that act as guide, stimulating and accelerating regrowth of the transected nerve and additionally forming a barrier to ingrowth of connective tissue (Faroni et al., 2015; Du et al., 2018).

To date, a wide variety of new synthetic polymers and biopolymers have been evaluated. Scaffolds of natural origins provide several advantages compared to the synthetic ones, such as biocompatibility, biodegradability, non-toxic degradation products and the minimal foreign body response induction (Carriel et al., 2014).

Silk fibroin (SF) is a natural polymer produced from the silkworm and it is one of the oldest materials used in medical applications. It is a highly biocompatible material known for its ability to promote cell adhesion and proliferation and to stimulate tissue regeneration *in vivo*, with a unique combination of biological and mechanical properties (Altman et al., 2003; Rockwood et al., 2011; Catto et al., 2015; Thurber et al., 2015).

We have recently developed a new conduit made by silk fibroin (SilkBridge^TM^ nerve conduit) consisting of a hybrid tubular structure composed by two electrospun layers (ES, made of regenerated silk fibroin fibers of sub micrometer size) coupled with an intermediate textile layer (TEX, made of native silk fibroin microfibers) (Alessandrino et al., 2019b). This novel multi-layered SF-based nerve conduit resulted in a perfectly integrated and mechanically resistant structure with a light weight and a high porosity level in the low pore size range, all features important for an optimal nerve conduit. In the previous work, we have demonstrated the biocompatibility and biomimeticity of SilkBridge^TM^ nerve conduit, both *in vitro* and *in vivo*. SilkBridge^TM^ nerve conduit was able to sustain Schwann cell proliferation, neuronal differentiation and axonal elongation *in vitro*. *In vivo* pilot tests conducted at 2 weeks post implantation revealed a perfect cellular colonization of the conduit and the progressive growth of the regenerating nerve fibers.

Given these promising results, in the current study we evaluated the efficiency of SilkBridge^TM^ nerve conduit in sustaining nerve regeneration at mid (4 weeks) and longer (12 and 24 weeks) time points, in a model of rat median nerve injury, using the autologous nerve repair approach as control.

## Materials and Methods

### Manufacturing of SilkBridge^TM^

The three-layered SF-based nerve conduit (SilkBridge^TM^) was manufactured as previously reported (Alessandrino et al., 2019a; Alessandrino et al., 2019b). Briefly, two electrospun layers (ES) were assembled onto the inner and outer faces of a tubular textile braid (TEX) according to a patented process (Alessandrino, 2017). Coupling of the TEX layer with the two ES layers was made during electrospinning, by means of a welding medium comprising a solution of 15% w/w SF dissolved in an ionic liquid (1-ethyl-3-methylimidazolium acetate; EMIMAc). After electrospinning, the hybrid ES-TEX tubular structure was consolidated by immersion in aqueous ethanol (80 vol%), followed by overnight washing with distilled water and drying. Finally, the device was purified by microwave assisted extraction with ethanol to remove processing aids, packaged under a laminar flow cabinet, and sterilized with ethylene oxide (EtO).

The main characteristics of the SilkBridge^TM^ conduits used in the present study are: total length of the device 30 mm (reduced for the *in vivo* implantation to a length of 12 mm); inner diameter 1.60 ± 0.15 mm; wall thickness 0.50 ± 0.15 mm; weight per unit length of about 8 mg cm^–1^; wall porosity of about 80%; ES:TEX percent weight ratio of 60:40%.

### Mechanical Characterization

Ultimate tensile strength and suture retention strength were determined on SilkBridge^TM^ conduits, under submersed conditions (in water at 37°C), by using an All-electric Dynamic Test Instrument ElectroPuls E3000 (Instron), equipped with a 250 N load cell and a thermostatic bath (BioPuls). Both tests were performed in accordance with the provisions of the ISO 7198:2016 standard, which specifies the requirements for the evaluation of the mechanical properties of prosthetic devices with tubular shape.

For the measurement of the ultimate tensile strength, six specimens with a total length of 50 mm were used. The gage length was 30 mm, a preload of 0.5 N was applied, and the tests were run at 50 mm min^–1^ crossbar rate.

The suture retention strength is the force necessary to pull a suture from the device while pulling a suture inserted through the wall. The conduit was cut normal to the long axis and a suture was inserted 2 mm from the end of the device through the wall to form a half loop. The device was clamped in the lower fixed grip and the suture thread in the upper moving grip which was pulled at the rate of 50 mm min^–1^. The force required to pull the suture through the device was recorded.

### Animal Care, Experimental Groups and Surgery

For this study, a total of 36 adult female Wistar rats (weight approximately 200 g) were used. Animals were housed in a room with controlled conditions (temperature and humidity), with a regular light/dark circle (12 h of light and 12 h of dark) and free access to food and water. Every attempt was made to reduce animal suffering. The study conditions were conformed to the guidelines of the European Union’s Directive EU/2010/63 for animal experiments. All animal experiments were performed at the animal facility of Neuroscience Institute Cavalieri Ottolenghi (NICO) (Ministerial authorization DM 182 2010-A 3-11-2010). The current experimental study was reviewed and approved by the Ethic Experimental Committee of the University of Turin (Ministry of Health project number 864/2016).

Analyses of nerve regeneration were carried out at three time points: 4, 12, and 24 weeks. The shortest time point (4 weeks) was planned with the aim to explore the outcome of middle endpoints, i.e., middle-stage tissue response to the conduit, extracellular matrix deposition, infiltration of Schwann cells, and axon regeneration. Animals (*n* = 4) with bilateral implantation of the SilkBridge^TM^ conduit were used. Medium-to-long term time points (12 and 24 weeks) were designed to follow the regeneration process until the steady state was achieved in terms of biological tissue response, healing of the injured nerve, and complete functional recovery. Animals (*n* = 10 for each time point) were implanted monolaterally with SilkBridge^TM^ conduits. Control animals (*n* = 6 for each time point) with autograft implants were also included in this part of the study.

Surgeries were performed under general anesthesia, with Zolazepam (Zoletil, Virban) + Xilazina (Bayer) by intraperitoneal injection (40 mg/kg +5 mg/kg). All surgical procedures were carried out under a high magnification surgical microscope, in a clean room. Nerve lesions were performed on the median nerves. The median nerve of both forelimbs of the 4 weeks experimental group was transected (10-mm gap) and a 12-mm long SilkBridge^TM^ conduit was used to bridge the nerve defect by inserting 1 mm of each nerve end inside the conduit. The nerve conduit was sutured with one 9/0 epineural stitch at each end (Figures 2A–D). SilkBridge^TM^ conduits were immersed in sterile saline for at least 5 min before implantation.

In all the other animals (12 and 24 weeks experimental and control groups), the median nerve of the right forelimb was approached from the axillary region to the elbow, the nerve was transected at the middle third of the brachium and its proximal stump was sutured with 9/0 epineural stitch to the *pectoralis major muscle* to avoid spontaneous reinnervation. Afterwards, the left median nerve was transected and immediately repaired according to the experimental group. For the SilkBridge^TM^ conduit group, the gap was bridged with a 12-mm long conduit as described above (Figures 2A–D). For the autograft group, the 10-mm nerve segment was cut out, reversed (distal – proximal), and sutured to the nerve ends with 9/0 epineural stitches. At the end of the surgical procedure, the skin was sutured with a 3/0 stich. At day 0, 1, 2, and 3 post-surgery, analgesic therapy with Rymadil (4 mg/Kg, Zoetis Italia) was administered by subcutaneous injection, while at day-1 pre-surgery, 2 and 5 post-surgery, the antibiotic treatment (Rubrocillina 0.05 ml/500 g, MSD animal health) was administered by intramuscular injection. The general health status of animals was evaluated by experienced personnel considering the following parameters: shine rat fur, reactivity, general health, and aspect of surgical wound. Animals were observed at day-7 pre-implantation, at day 0 before surgery, and at day 1, 2, and 3 post-surgery. From the 2nd week after surgery weekly routine monitoring was carried out regularly. Weight evolution of animals was also recorded at day-1 and every 3 weeks post-surgery. The last observation coincided with the last day of procedure.

The last day of procedure rats were sacrificed through anesthetic overdose of Zoletil + Xilazina (>60 and >10 mg/kg) by intraperitoneal injection. The surgical site was exposed, and the nerve samples were harvested and processed for further examination. The *superficialis flexor muscles* were harvested and weighted.

### Evaluation of Nerve Regeneration After 4 Weeks: Histological Procedures

Four nerve samples harvested after 4 weeks were fixed in 4% paraformaldehyde for 2 h, washed in a solution of 0.01 M PBS (pH 7.2) for 30 min. For Crio-embedding procedure, specimens were rehydrated with PBS (Sigma) and cryo-protected with three passages in increasing solutions of sucrose (Sigma) (7.5% for 1 h, 15% for 1 h, 30% overnight) in 0.1 M PBS. Thereafter, specimens were maintained in a 1:1 solution of sucrose 30% and optimal cutting temperature medium (OCT, electron microscopy sciences) for 30 min and then embedded in 100% OCT. Sections were cut 10 μm thick and processed for Masson’s trichrome staining or immunofluorescence. The other four nerve samples were fixed by immediate immersion in 2.5% glutaraldehyde (SIC, Società Italiana Chimici) in 0.1 M phosphate buffer (pH 7.4) for 5.6 h, at 4°C and subjected to resin embedding, high resolution light microscopy and transmission electron microscopy.

#### Masson’s Trichrome Staining

Masson’s trichrome staining was performed on cryo-embedded longitudinal section according to Masson trichrome with aniline blue kit (Bio-Optica). After staining, slides were washed in distilled water, rapidly dehydrated in ethanol and cleared in xylol/Bioclear (Bio-Optica). Finally, samples were mounted with DPX mountant (Fluka).

#### Immunofluorescence and Confocal Laser Microscopy

Crio-embedded longitudinal sections were permeabilized, blocked with 0.1% triton X-100, 10% normal goat serum for 1 h and incubated overnight with the primary antibodies anti-NF 200 kDa (monoclonal, mouse, Sigma Aldrich) and S-100 (polyclonal, rabbit, Sigma Aldrich). After primary antibodies incubation, sections were washed three times in PBS and incubated for 1 h in a solution containing the secondary antibodies Alexa 488 anti-Mouse and Cy3 anti-Rabbit (Life Technologies). Nuclei were stained with 4,6-diamidino-2-phenylindole (DAPI, Sigma) diluted 1:1000 in PBS. After three washes in PBS, sections were mounted with a Dako fluorescent mounting and analyzed using a Zeiss LSM800 confocal laser microscopy system (Zeiss, Jena, Germany).

#### High Resolution Light Microscopy and Transmission Electron Microscopy

The nerve samples fixed in 2.5% glutaraldehyde were post-fixed in 2% osmium tetroxide (SIC, Società Italiana Chimici) for 2 h and dehydrated in passages in ethanol (Sigma Aldrich) from 30 to 100% (5 min each passage). After two passages of 7 min in propylene oxide, one passage of 1 hour in a 1:1 mixture of propylene oxide (Sigma Aldrich) and Glauerts’ mixture of resins, samples were embedded in Glauerts’ mixture of resins (made of equal parts of Araldite M and the Araldite Harter, HY 964, Sigma Aldrich). In the resin mixture, 0.5% of the plasticizer dibutyl phthalate (Sigma Aldrich) was added. For the final step, 2% of accelerator 964 was added to the resin in order to promote the polymerization of the embedding mixture, at 60°C. Transverse semi-thin sections (2.5 μm thick) were cut inside the conduit (both proximally and distally) using an Ultracut UCT ultramicrotome (Leica Microsystems, Wetzlar, Germany) and stained with 1% toluidine blue for high resolution light microscopy using a DM4000B microscope equipped with a DFC320 digital camera. Ultra-thin sections (70 nm thick) were cut with the same ultramicrotome. Sections were analyzed using a JEM-1010 transmission electron microscope (JEOL, Tokyo, Japan) equipped with a Mega-View-III digital camera and a Soft-Imaging-System (SIS, Münster, Germany) for the computerized acquisition of the images.

### Evaluation of Nerve Regeneration After 12 and 24 Weeks

#### Grasping Test

The grasping test on 12 and 24 weeks experimental and control groups was performed every 3 weeks. The last observation coincided with the last day of procedure. The aim of the test was to evaluate the functional recovery of the operated nerve by assessing the flexor muscle strength. The animal was gently lifted by holding its tail and allowing it to grasp a grid connected to an electronic balance (BS-GRIP Grip Meter). The quantitative assessment was made by measuring the maximum weight that the rat was able to hold before losing its grip. Each animal was tested three times and the average was considered.

#### Quantitative Assessment of Myelinated Regenerated Nerve Fibers

Transverse semi-thin sections (2.5 μm thick) were cut distally to the conduit/autograft and stained with 1% toluidine blue for high resolution light microscopy examination and design-based stereology. A DM4000B microscope equipped with a DFC320 digital camera and an IM50 image manager system (Leica Microsystems, Wetzlar, Germany) was used for section analysis. For the stereological analysis, the following parameters were evaluated: (i) number of fibers; (ii) density of fibers; (iii) diameter of fibers and axons; (iv) myelin thickness; and (v) axon diameter/fiber diameter ratio (g-ratio). Sections were randomly selected and analyzed for the measurement of the total cross-sectional area of the nerve. The stereological assessment was performed according to a previously described method (Geuna, 2000; Geuna et al., 2000). 2D dissector probes were also used to select unbiased representative samples of myelinated nerve fibers.

### Assessment of Biomaterial Behavior After Implantation

#### Analysis of New-Generated Vessels

The process of angiogenesis within the SilkBridge^TM^ conduits was assessed by quantifying new blood vessel formation in resin-embedded transverse semi-thin sections. On one randomly selected semi-thin section taken in the central portion of the conduit, 8–10 fields were selected using a systematic random sampling protocol, with a magnification of 40×. The two-dimensional dissector procedure was adopted for the quantification (Geuna, 2000). Blood vessel density was then calculated. Finally, the diameter of the vessels was measured, and the vessel diameter distribution was obtained.

#### Biomaterial Degradation

SilkBridge^TM^ conduit degradation was evaluated both qualitatively and quantitatively. For qualitative analysis, the behavior of the conduit wall (consisting in three layers: inner and outer electrospun layers and middle textile layer) was carefully analyzed in semi-thin cross sections in order to observe and describe any variation compared to the non-implanted conduit.

Quantitative analysis of conduit degradation was focused on the two electrospun layers (inner and outer), since they are in direct contact with surrounding tissue (outside) and regenerating nerve fibers (inside). These two lavers are made by silk fibroin fibers of sub micrometer size, and the analysis was therefore done with the transmission electron microscopy on ultra-thin cross sections. 15–20 fields were selected using a systematic random sampling protocol, with a magnification of 20000×, and a total of 350 fibers for each group were measured. As control, we used SilkBridge^TM^ conduits implanted for 2 weeks.

### Statistical Analysis

Statistical analysis was performed using R Statistical Software (Foundation for Statistical Computing, Vienna, Austria). After data normality was tested (Levene and Mauchly tests), one-way analysis of variance (ANOVA) and ANOVA for repeated measures tests with Tukey’s correction were adopted to detect the effect of time, experimental groups, and their interaction and to highlight the significant differences among the Autograft group and the SilkBridge^TM^ group at each time point tested. Two one-sided tests (TOST) equivalence test was used to assess the effect of the two different interventions (Supplementary Material). The Cohen’s *d* obtained between beginning and end values of the Autograft group was adopted as the smallest effect size and set as a reference for the TOST test. The two sided paired Student’s T test was adopted to compare data on vessel morphology and 95% Confidence Intervals (CI) were reported (Mascha and Sessler, 2011). The effect size was defined for each factor as partial eta-squared (η^2^) small 0.02, medium 0.13 and large 0.26. The level of significance was set at *p* ≤ 0.05 (^∗^), *p ≤* 0.01 (^∗∗^), *p ≤* 0.001 (^∗∗∗^), and *p ≤* 0.000 (^****^). Values were expressed as mean ± SD (standard deviation).

## Results

### Mechanical Characterization

**FIGURE 1:**
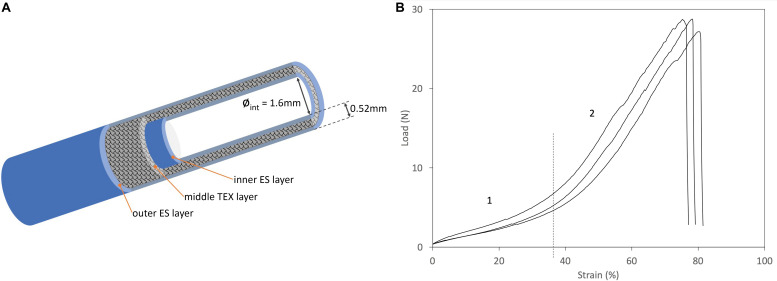
**(A)** Schematic diagram of the 3D architecture of the SilkBridge^TM^ conduit. **(B)** Typical load/strain curves. Phase 1: Low-load region, corresponding to the “toe region” of the load-elongation curve of natural nerves (Kwan et al., 1992). Phase 2: Steady rate of elongation region.

The mechanical characterization discussed in this paper complements the results previously reported (Alessandrino et al., 2019a; Alessandrino et al., 2019b) which referred specifically to the behavior of the conduit subjected to transversal stresses in the compression mode. Here, the mechanical properties of the SilkBridge^TM^ conduit are investigated in the longitudinal direction, by testing the ultimate tensile strength and the suture retention strength. The results of tensile and suture retention strength are listed in Supplementary Table S1. Figure 1B shows typical load-elongation curves of the SilkBridge^TM^ conduit measured under submersed condition at 37°C. A schematic representation of the 3D architecture of the conduit is also presented in Figure 1A.

The SilkBridge^TM^ conduit breaks at high strength values when subjected to a force applied in the longitudinal direction, thanks to its hybrid electrospun/textile architecture (Supplementary Table S1). The braided TEX structure encased between the two ES layers is the load-bearing component of the device (Figure 1A). In terms of ultimate tensile strength, SilkBridge^TM^ outperforms not only natural peripheral nerves (Chiono and Tonda-Turo, 2015), but also many other nerve conduit devices made only by electrospun fibers, either based on SF (Dinis et al., 2015) or on other polymers such as PLGA (Hou et al., 2019), PCL (Quan et al., 2019), and PU/gelatin (Salehi et al., 2018). With reference to other nerve conduits with a braided texture made of native SF microfibers (Pillai et al., 2019) or other polymer fibers (PLA/PGA) (Ichihara et al., 2015), the mechanical performance may be similar or better depending on the construction parameter of the textile structure (yarn and fiber size, knit density, etc.). Usually, the denser and thicker the textile texture, the stiffer the device, which might become a drawback in terms of biomechanical compliance at the site of implantation. In fact, when a load is first applied to a resting natural nerve, its length increases with minimal increase of the tensile load as a result of straightening of the wavy connective tissue and axons in the endoneurial compartment (Topp and Boyd, 2006). This is called the “toe region” of the load-elongation curve of natural nerves (Kwan et al., 1992). As the tensile load is further increased, the nerve elongates at a steady rate, showing a steeper linear region of the load-elongation curve before ultimate failure. Interestingly, the combination of two electrospun layers with an open-texture textile layer of braided silk microfibers results in a load-elongation curve with two distinct phases (Figure 1B) that mimics the tensile behavior of natural nerves (Kwan et al., 1992), as well as of other soft tissues (Holzapfel and Weizsacker, 1998). Therefore it is possible to deduce that the SilkBridge^TM^ conduit, which displays a balanced combination of strength and elasticity, might be more biomechanically compliant with natural nerves than other devices where a much higher ultimate tensile strength was reached at the expenses of a dramatic loss of elasticity, especially in correspondence of the “toe region” of the natural nerve (Zhang et al., 2019).

The hybrid electrospun/textile structure of SilkBridge^TM^ is also beneficial for the achievement of high values of suture retention strength (Supplementary Table S1). With the 9/0 suture, the same used for implanting the device in the animal study, the test ends with the failure of the suture at about 117 gf (Supplementary Table S1). Failure of the device occurred at about 460 gf using a thicker suture stich (5/0). These results confirm that the suturing process can be easily and safely performed during the grafting surgery, and that failure after surgery can be reasonably considered an unexpected event for the SilkBridge^TM^ conduit.

### Assessment of Medium and Long-Term Nerve Regeneration

#### Surgical Procedures, Animal Welfare, and Macroscopic Evaluations

As already demonstrated in a previous work (Alessandrino et al., 2019b) SilkBridge^TM^ showed optimal mechanical parameters during surgery, including easy handling and suturability, and adequate stiffness and flexibility. During post-operative period, all animals were in good health. None of them showed signs of inflammation, pain, discomfort or auto-mutilation of the operated arm. As an indicator of animal welfare, the body weight was measured. No sudden decrease in weight was observed and animals showed a physiological increase in body mass throughout the experiment (data not shown).

**FIGURE 2:**
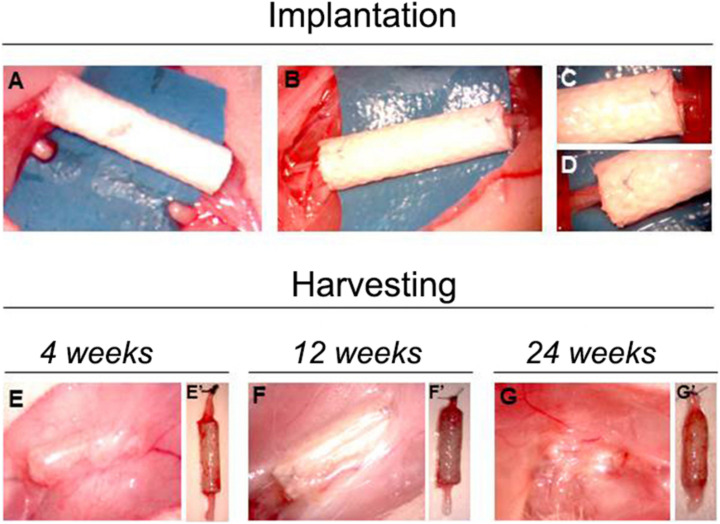
Pictures showing the implantation and the harvesting surgeries. Panels **(A–D)** show the phases of SilkBridge^TM^ implantation, from the creation of the median nerve gap **(A)** to the position and suturing of the conduit **(B–D)**. **(E–G)** Show the macroscopical evaluation of SilkBridge^TM^ conduit during harvesting. **(E–G)** SilkBridge^TM^ implantation site exposed before the harvesting at different time points. No signs of inflammation can be detected; **(E’–G’)**: SilkBridge^TM^ harvested at different time points; surgical stich marks the proximal stump.

At the time of sample harvesting, all conduits were still clearly recognizable. They were encapsulated in a thin layer of connective tissue. No signs of inflammation, foreign body reactions or scar tissue formation around the conduit was detected, confirming the good biocompatibility of SilkBridge^TM^ (Figures 2E–G).

#### Medium-Term Nerve Regeneration

**FIGURE 3:**
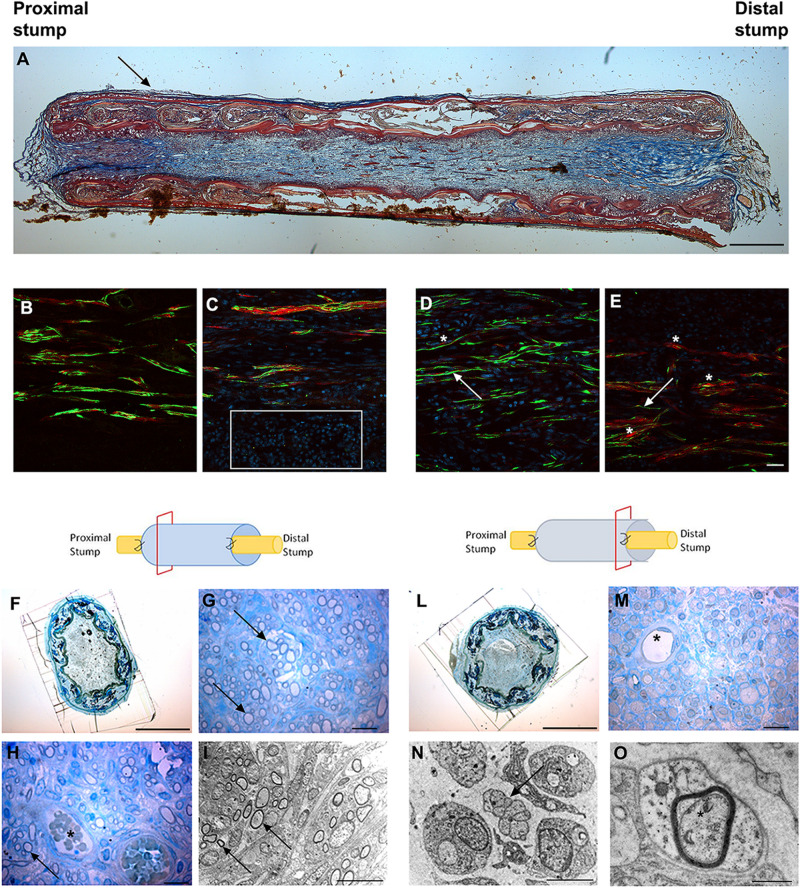
Images showing the morphological evaluation of nerve regeneration inside SilkBridge^TM^ nerve conduit at 4 weeks. **(A)** Representative longitudinal section of repaired median nerve analyzed by Masson’s trichrome staining. Black arrow indicates the layer of connective tissue. Scale bar: 1000 μm. **(B–E)** Representative immunofluorescence staining performed on proximal and central part of SilkBridge^TM^ conduit. Scale bar: 20 μm; **(B)** Nerve fibers and Schwann cells in the proximal stump; **(C)** Several nuclei (DAPI-blue, white box) and regenerated nerve fibers localized within the conduit; **(D,E)** Positive neurofilament fibers surrounded by S-100 positive Schwann cells in the center of the conduit. White arrows indicate nerve fibers (green); asterisk indicate the Schwann cells (red). **(F–H)** Representative high resolution light micrographs of toluidine blue-stained semi-thin proximal cross sections; **(G,H)** Black arrows indicate regenerated nerve fibers in the proximal part of the conduit, while blood vessels are marked by asterisks. **(I)** Representative electron microscopy images of regenerated median nerve (nerve fibers black arrows) taken inside the grafts, proximally. **(L,M)** Representative high resolution light micrographs of toluidine blue-stained semi-thin distal cross sections; **(N,O)** Representative electron microscopy images of regenerated median nerve taken inside the grafts, distally, at different magnifications. Black arrows underlie the presence of unmyelinated fibers, the asterisk marks the presence of a myelinated fiber with a thin myelin sheath. **(H–M)** Asterisk indicates blood vessels. Scale bar **(F,L)**: 1000 μm; Scale bar **(G,H,M)**: 20 μm; Scale bar **(I,N)**: 10 μm; Scale bar **(O)**: 1 μm.

We observed the nerve morphology in the medium period of regeneration (4 weeks post-surgery) using histological staining, immunohistochemical labeling and electron microscopy analysis (Figure 3).

Longitudinal sections stained with Masson’s trichrome staining revealed a thin layer of connective tissue surrounding the outer side of SilkBridge^TM^ and the absence of scar tissue formation. Moreover, a reach cellular population colonizing the full length of the conduit was observed (Figure 3A).

Immunohistochemical examination revealed many neurofilament positive regenerated nerve fibers surrounded by S-100 positive Schwann cells, especially in the proximal and mid portion of the conduit (Figures 3B–E) indicating the progression of nerve regeneration throughout the conduit.

In the proximal portions of the conduit, semi-thin toluidine blue-stained transverse sections revealed many well-myelinated axons (Figures 3G,H), visualized also through electron microscopy analysis (Figure 3I). In the distal portions of the conduit, semi-thin toluidine blue-stained transverse sections did not allow the visualization of regenerated myelinated nerve fibers (Figure 3M). However, electron microscopy analysis revealed numerous unmyelinated fibers and few regenerating fibers with a thin myelin sheath, indicating that the myelination process was still in progress in the distal portion of the conduit (Figures 3N,O).

Finally, many blood vessels, some of them with big diameter, were detected not only in the lumen of the conduit (Figures 3H–M) but also among the layers of the wall throughout the full length of the conduit (not shown).

#### Long-Term Nerve Regeneration

The effectiveness of SilkBridge^TM^ in stimulating nerve regeneration was then evaluated at long-term time points (12 and 24 weeks) and was compared to the “gold standard” technique (Autograft).

**FIGURE 4:**
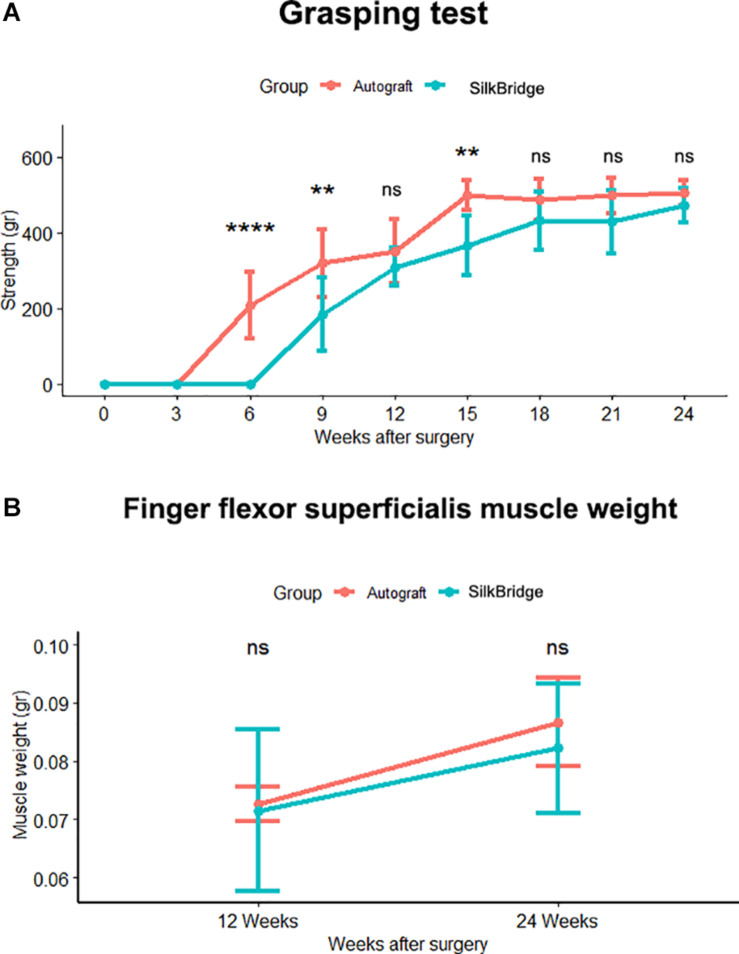
**(A)** *In vivo* functional analysis. Line graph reporting the post traumatic time course of functional recovery assessed by the grasping test. Significant difference, tested through ANOVA for repeated measures with Tukey’s correction, in functional recovery between SilkBridge^TM^ and autograft group was observed at 6, 9, and 15 weeks. (^∗^) *P* ≤ 0.05, (^∗∗∗^) *P* ≤ 0.000. **(B)** Line graph depicting the *Finger flexor superficialis muscle* weight. No statistical significance was present between groups at each time point. Values are reported as mean ± Standard Deviation. Group 12 weeks *n*: 16 (SilkBridge^TM^), 10 (Autograft); Group 24 weeks *n*: 8 (SilkBridge^TM^), 5 (Autograft).

Functional recovery was investigated starting from 3 weeks until 12 or 24 weeks by means of the grasping test. The graph in Figure 4 (Figure 4A) reports the post-traumatic time course of functional recovery. The function of the finger flexor muscles innervated by the median nerve started recovering faster in the Autograft group, as indicated by the statistically different performance recorded at weeks 6 (*p* < 0.000) and 9 (*p* < 0.001) after lesion. Animals implanted with SilkBridge^TM^ started recovering at weeks 6 and then progressively improved. With exception of the weeks 15 (*p* < 0.002) time point, where SilkBridge^TM^ and Autograft groups still showed a statistically significant difference, at 12 weeks (*p* = 0.105) and then from 18 weeks (*p* = 0.152) until the end of the test, no significant differences were possible to be observed between the two experimental groups (21 weeks: *p* = 0.086; 24 weeks: *p* = 0.153). The within subjects test revealed a significant effect for time (*F*[8,196] = 262.91, *p* < 0.000, η^2^ = 0.91), treatment (*F*[1,196] = 85.11, *p* < 0.000, η^2^ = 0.30) and time x group (*F*[8,196] = 9.64, *p* < 0.000, η^2^ = 0.28) factors.

As an additional indicator of motor recovery, the forelimb *finger flexor superficialis muscles* were harvested from both SilkBridge^TM^ and Autograft animals and their fresh weight was determined (Figure 4B). The weight of the muscles increased significantly from 12 to 24 weeks (*F*[1,26] = 9.997, *p* < 0.003, η^2^ = 0.28), but no significant differences were observed between the two groups (*F*[1,26] = 0.507, *p* < 0.483, η^2^ = 0.02) and within time (*F*[1,26] = 0.183, *p* < 0.673, η^2^ = 0.01). This result that underscores the good performance of animals that underwent nerve reconstruction by means of the SilkBridge^TM^, is in good agreement with the trend evidenced by functional recovery tests.

**FIGURE 5:**
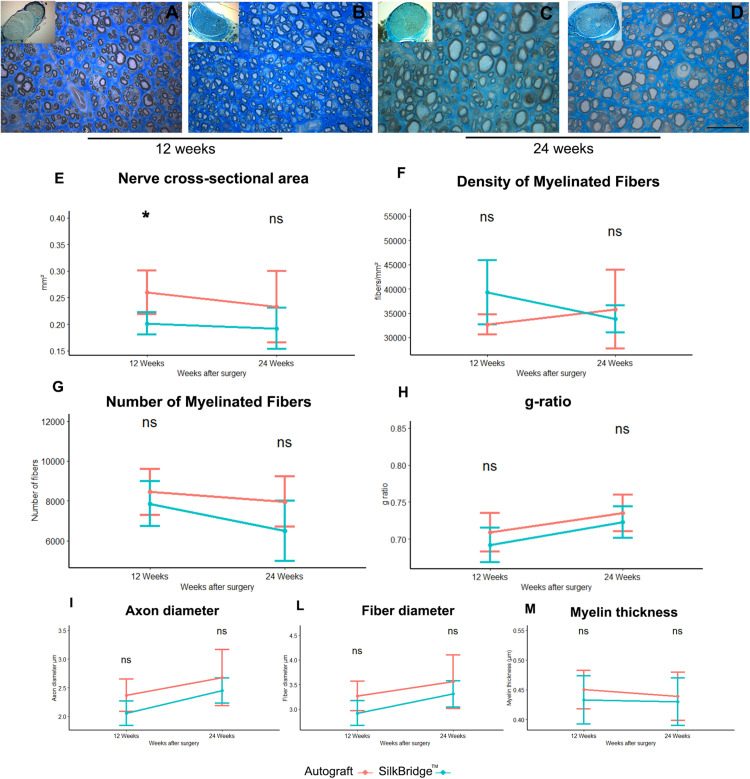
Morphological and morphoquantitative evaluation of nerve regeneration after 12 and 24 weeks. **(A–D)** Representative low and high magnification light photomicrographs of toluidine blue–stained semi-thin cross sections of regenerated nerve using autograft **(A,C)** or SilkBridge^TM^ nerve conduit **(B,D)** obtained distally to the graft, at 12 **(A,B)** and 24 **(C,D)** weeks after surgery. Scale bar: 20 μm. **(E–M)** Stereological assessment of regenerated nerve fibers of autograft and SilkBridge^TM^ groups. **(E)** Nerve cross-sectional area; **(F)** Density of myelinated fibers; **(G)** Total number of myelinated nerve fibers; **(H)**: g-ratio (axon diameter/fiber diameter); **(I–M)**: parameters related to the size: axon diameter, fiber diameter and myelin thickness. Data are expressed as mean ± Standard Deviation. Asterisks (^∗^) denote statistically significant differences between autograft and SilkBridge^TM^ conduit group at each time point (12 and 24 weeks experimental time points); (^∗^) ≤ 0.05. Group 12 weeks *n*: 8 (SilkBridge^TM^), 5 (Autograft); Group 24 weeks *n*: 8 (SilkBridge^TM^), 5 (Autograft).

Stereological and morpho-quantitative analysis of regenerated nerve fibers were performed on toluidine blue–stained semi-thin cross sections, just distally to the graft. At 12 weeks after nerve reconstruction, regenerated nerves from SilkBridge^TM^ and Autograft groups showed many re-growing myelinated fibers organized in microfascicles, with well-defined axoplasm and well-organized myelin sheaths (Figures 5A–D). Quantitative stereological analysis of semi-thin sections revealed that the cross-sectional area of the nerve regenerated (Figure 5E) decreased but not significantly from 12 to 24 weeks (*F*[1,22] = 0.971, *p* = 0.335, η^2^ = 0.04), inside the SilkBridge^TM^ conduit (and measured distally to the conduit). It was significantly smaller than that of the Autograft group (*F*[1,22] = 8.745, *p* < 0.007, η^2^ = 0.28) but, no significant differences were observed between the two groups and within time (*F*[1,22] = 0.278, *p* < 0.604, η^2^ = 0.01).

The density of myelinated fibers was similar in both groups (Figure 5F) and no effect of time (*F*[1,22] = 1.057, *p* = 0.315, η^2^ = 0.05), treatment (*F*[1,22] = 1.116, *p* = 0.302, η^2^ = 0.05) and time by treatment was significant (*F*[1,22] = 3.947, *p* = 0.060, η^2^ = 0.15).

The total number of myelinated fibers showed no significant difference between the two groups (*F*[1,22] = 3.933, *p* = 0.060, η^2^ = 0.152) and no time (*F*[1,22] = 4.096, *p* = 0.055, η^2^ = 0.157) nor time by treatment effect was detected (*F*[1,22] = 0,714, *p* = 0.407, η^2^ = 0.031; Figure 5G). The highest density of myelinated fibers in the Autograft group of about 1033.26 n/fibers was not significantly superior to the SilkBridge^TM^ conduit group (*p* = 0.061, 95% CI: −47.20–2113.73).

Noteworthy, g-ratio (Figure 5H), one of the more reliable morphological predictors of nerve recovery, was significantly different between the two time points (*F*[1,22] = 9.978, *p* < 0.005, η^2^ = 0.312) but, no significant effect was detectable for type of intervention (*F*[1,22] = 2.440, *p* = 0.133, η^2^ = 0.10) and intervention by time factor (*F*[1,22] = 0.075, *p* = 0.787, η^2^ = 0.003). The mean difference of g-ratio between Autograft and SilkBridge^TM^ conduit group of about 0.001 is not significant (*p* = 0.132, 95% CI: −0.01–0.03).

After 24 weeks, the regenerating nerves continued their maturation process in terms of fiber size and myelin organization, as evidenced by the histological details (Figures 5C,D). The stereological parameter that were significantly different at 12 weeks between SilkBridge^TM^ and Autograft groups (nerve cross-sectional area) resulted to be similar at 24 weeks (Figure 5E). Futhermore, myelin thickness was not affected by treatment (*F*[1,22] = 0.409, *p* = 0.7, η^2^ = 0.007), time (*F*[1,22] = 0.152, *p* = 0.7, η^2^ = 0.007) or interaction between those factors (*F*[1,22] = 0.068, *p* = 0.797, η^2^ = 0.003). The mean difference of myelin thickness between Autograft and SilkBridge^TM^ conduit group of about 0.013 is not significant (*p* = 0.408, 95% CI: −0.02–0.05). Considering the fiber diameter a significant effect was detected for time and treatment factors (*F*[1,22] = 7.125, *p* < 0.014, η^2^ = 0.245; and *F*[1,22] = 4.864, *p* = 0.038, η^2^ = 0.181) but not for their interaction (*F*[1,22] = 0.138, *p* = 0.713, η^2^ = 0.006). The fiber diameter in the Autograft group was significantly superior to the SilkBridge^TM^ group of about 0.298 μm (p < 0.038, 95% CI: −0.02 – 0.05). Referring to the axons diameter a significant effect was detected for time and treatment factors (*F*[1,22] = 9.739, *p* < 0.005, η^2^ = 0.307; and *F*[1,22] = 5.159, *p* = 0.033, η^2^ = 0.190) but not for their interactions (*F*[1,22] = 0.124, *p* < 0.728, η^2^ = 0.006). The axon diameter in the Autograft group was significantly superior to the SilkBridge^TM^ group of about 0.271 μm (*p* < 0.024, 95% CI: 0.02–0.052).

### Biomaterial *in vivo* Long-Term Implantation

#### Morphometrical Analysis of New-Generated Vessels

**FIGURE 6:**
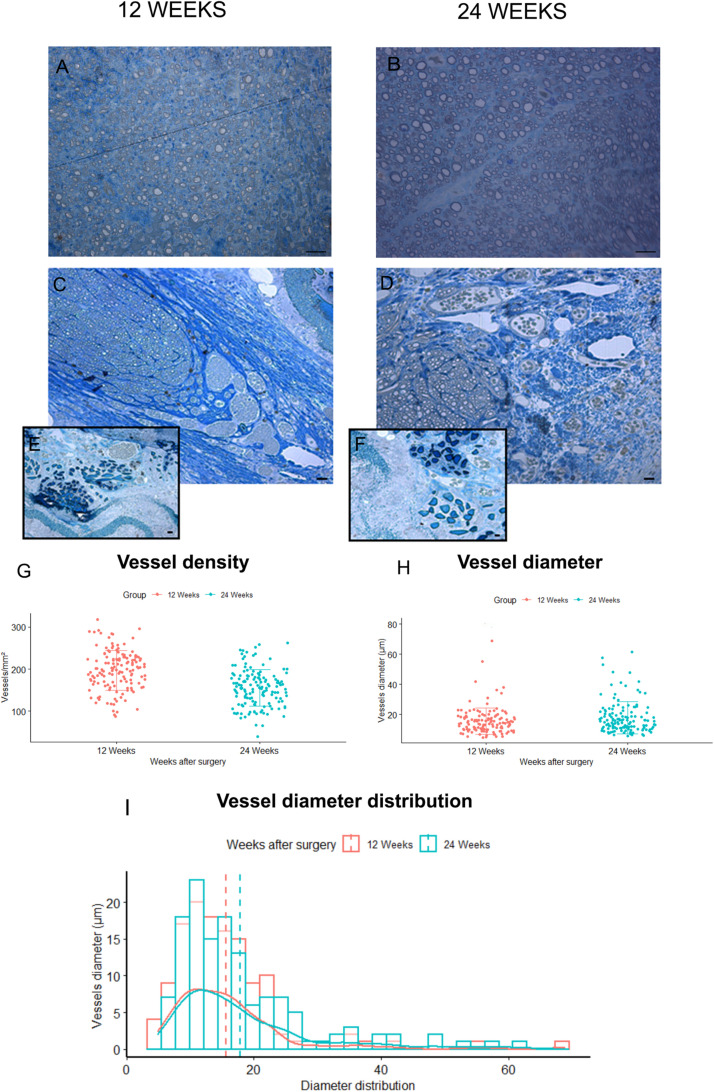
Blood vessels analysis. **(A,B)** Representative toluidine blue–stained semi-thin cross sections of Autograft harvested at 12 **(A)** and 24 **(B)** weeks: only microvessels and capillaries of small dimensions are visible. **(C,D)** representative toluidine blue–stained semi-thin cross sections of SilkBridge^TM^ harvested at 12 **(C)** and 24 **(D)** weeks: many vessels of different size are easily recognized at the periphery of the regenerated nerves and between the three layers of the wall **(E,F)**. Scale bars: 20 μm. **(G–I)** Stereological assessment of newly formed blood vessels in SilkBridge^TM^ long-term groups; Box Plot depicting: **(G)** vessels density, **(H)** vessels diameter. Bar graph representing vessel diameter distribution **(I)**. Data are expressed as mean ± Standard Deviation. Group 12 weeks *n*: 3 (SilkBridge^TM^), Group 24 weeks *n*: 3 (SilkBridge^TM^).

The morphological aspect of the regenerated tissue inside the grafts was assessed through toluidine blue–stained semi-thin cross sections obtained from the mid-portion of SilkBridge^TM^ experimental groups (Figures 6C–F). Micrographs at low magnifications (Figures 6C,D) showed that regenerated myelinated axons grown inside SilkBridge^TM^ were organized and packed in the central part of the conduit. Extracellular matrix, cells and blood vessels colonized the portion between the wall of the conduit and the regenerated fibers. On the other hand, the whole cross-section of the Autograft was full of regenerated fibers (Figures 6A,B).

Regarding blood vessels in the SilkBridge^TM^ experimental groups, a quantitative analysis was conducted in order to estimate their density and size (Figures 6G–I). In particular, blood vessel with a diameter bigger that 7 μm (and therefore easily recognizable in toluidine blue–stained sections) were considered. It has been observed with interest that, in the two regenerative timepoints examined a large number of newly formed blood vessels were found in SilkBridge^TM^ (Figures 6G–I). Despite no significant differences were detected between 12 and 24 weeks (Figures 6G,H) considering vessels density (*t*[1,135] = 7.62, *p* = 0.063, 95% CI: −52.4 – −31.2) and diameter (*t*[1,135] = 0.47, *p* = 0.068, 95% CI: −0.16 – 4.58) a slight tendency to vessel maturation was observed over time (Figure 6H). Their localization was particularly found at the periphery of the regenerated tissue, very close to the wall (Figures 6C–D) and in addition, blood vessels of varying dimensions were also found between the three layers of the conduit wall (Figures 6E,F). No vessels of this dimension were found in Autograft groups, only microvessels and capillaries of small dimensions (Figures 6A,B).

#### Biomaterial Degradation

The degradation of SilkBridge^TM^was evaluated qualitatively by high resolution light microscopy and quantitatively by transmission electron microscopy. The morphological analysis was performed in order to highlight the interaction between the conduit, the cells, and the extracellular matrix as well as to evidence any discontinuity of its original shape and structure; the stereological analysis was performed to evaluated changes in electrospun fibers diameter overtime. The 2 week timepoint, the shortest post-operative timepoint tested *in vivo* and described in Alessandrino et al. (2019b), was chosen as control for the comparison of degradation. This allowed to observe a sample not yet degraded but bearing some structural modifications caused by the *in vivo* environment.

**FIGURE 7:**
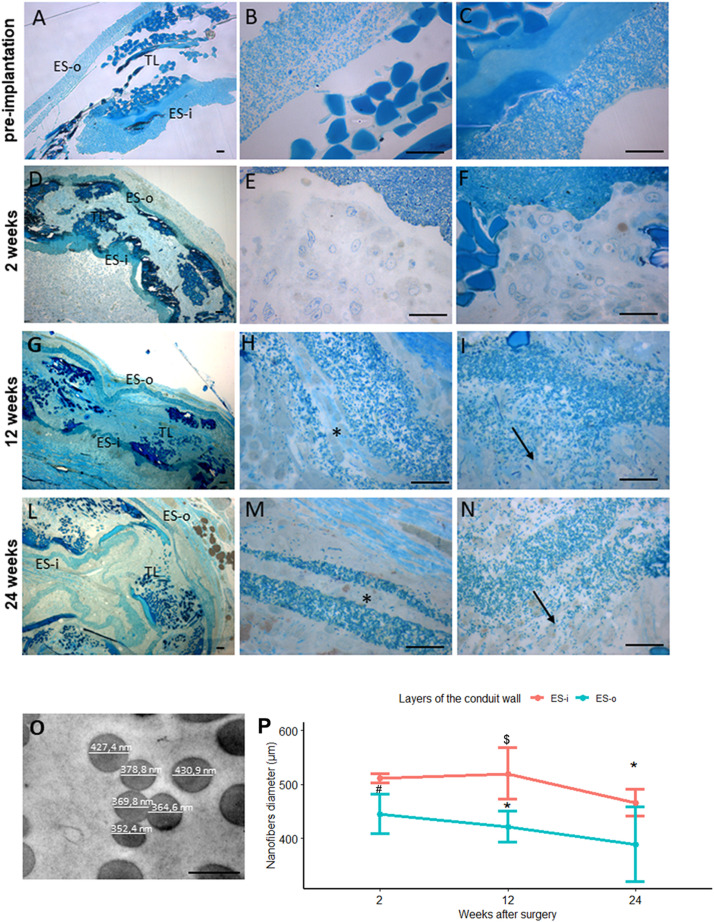
Evaluation of biomaterial degradation. **(A–N)** Representative high resolution light photomicrographs of toluidine blue–stained semi-thin cross sections showing the behavior of the SilkBridge^TM^ conduit wall at different time points. **(A–C)** SilkBridge^TM^ not implanted; **(D–F)** SilkBridge^TM^ implanted for 2 weeks, **(G–I)** for 12 weeks and **(L–N)** for 24 weeks. **(A,D,G,L)**: low magnification showing the three layers of SilkBridge. Scale bars: 20 μm; **(B,E,H,M)**: magnification of the outer ES-o layer. Scale bars: 20 μm; **(C,F,I,N)**: magnification of the inner ES-i layer. Scale bar: 20 μm; **(O)**: representative image of electrospun fibers of the ES layer with diameter measurements. Scale bar: 0,5 μm. ES-o: ES outer layer; ES-i: ES inner layer; TL: TEX layer. (**H,M**-asterisks) the ES outer layer that in some cases divides in two thinner layers; (**I,N**-arrows) the electrospun fibers dispersion in the ES inner layer. **(P)** Line graph depicting the quantification of the electrospun fiber diameter constituting the external ES wall (ES-o) and the internal ES wall (ES-i) of the SilkBridge conduit. Data are expressed as mean ± Standard Deviation. Significant differences within each time point are reported (^∗^)*P* ≤ 0.05. $ indicates the statistically significant difference in fibers diameter of inner ES layer between 12 and 24 weeks; ^#^Indicates the statistically significant difference in fibers diameter of outer ES layer between 2 and 12 weeks. *n*:350 fibers analyzed for each experimental group.

In non-implanted SilkBridge^TM^ the three layers of the conduit wall (the outer-ES-o, the inner-ES-i and the middle textile-TEX layer) are well defined and organized (Figure 7A); at higher magnification more details are detectable: the two ES layers are homogeneous and packed with TEX in the middle (Figures 7B,C).

After 2 weeks of nerve repair through SilkBridge^TM^ conduit the wall structure is still conserved: at low magnification a thin layer of connective tissue is visible on the ES-o layer suggesting an integration of the conduit with the surrounding tissue (Figure 7D); at higher magnification many cells and extracellular matrix colonized both the ES-o and ES-i layers (Figures 7E,F). After 12 weeks of implantation, a morphological integrity of the structure (Figure 7G) can still be appreciated at low magnification. Interestingly, at higher magnification, large portions of the ES-o layer appeared still compact, whereas more boundary regions started losing their compactness with extracellular matrix growing between the electrospun fibers sub-layers (Figure 7H). The same is detectable on the ES-i, proving an onset of degradation with variable intensity, with flaked areas at different degrees (Figure 7I). After 24 weeks, the ES-o layer was still continuous, displaying regions of high compactness, and others with flaked ES-o sub-layers forming two or more arrays of thinner layers (Figure 7L). At higher magnification, electrospun fibers at the outer limit of the ES-o layer progressively lost contact with each other. The extracellular matrix filled the inter-fiber areas, showing only a slight progression compared to the 12 weeks-time point (Figure 7M). On the contrary, the ES-i layer underwent greater modification since discontinuities along its circumferential path were observed in various regions. Moreover, the initial layer compactness was sensibly lost and individual electrospun fibers were fully embedded in the extracellular matrix showing a full integration of the material with the regenerated tissue (Figure 7N).

The conduit degradation analysis was continued with the quantification of the electrospun fibers diameter of the inner and outer ES layers at 2, 12, 24 weeks post implantation (Figure 7O).

Results showed a reduction of the SilkBridge^TM^ electrospun fibers diameter of both inner and outer ES layers overtime. In particular the inner ES layer had shown a significant difference between 12 and 24 weeks (*t*[1,30] = 3.87, *p* < 0.01) and the outer ES layer between 2 and 12 weeks (*t*[1,30] = 3.25, *p* < 0.03). Those data are in complete agreement with what was observed in the morphological analysis (Figure 7P).

## Discussion

Despite the intrinsic ability of peripheral nerve to regenerate, clinical and experimental evidence shows that regeneration is often unsatisfactory especially following severe nerve injury (Navarro et al., 2007). For lesions with loss of substance, the nerve autograft represents the surgical gold standard, despite the secondary effects of this technique (additional surgery, scarring, donor-site morbidity, and limited source of donor nerves) (Battiston et al., 2009).

Over the past ten years, advances in tissue engineering have led to obtain decellularized nerve allografts (Philips et al., 2018; Chato-Astrain et al., 2020) which allow to maintain the three-dimensional structure to sustain axonal growth, while being cleaned of the antigenic component. Recent papers reported the advantages of this technique and the achievement of some regenerative parameters obtained with allograft (Lovati et al., 2018; Chato-Astrain et al., 2020). However, further research is needed to optimize preparation protocols, improve effectiveness, especially for the repair of long nerve defects.

In parallel, a wide variety of new synthetic polymers and biopolymers have been evaluated. Scaffolds of natural origins are able to provide biocompatibility, biodegradability, non-toxic degradation products and a minimal foreign body response induction (Carriel et al., 2014). Authors have reported of biologically active devices whose lumen has been enriched through nanostructures and/or stem cells, capable of directing axonal growth and providing trophic factors and molecules in support of mechanical cues (Chato-Astrain et al., 2018).

When a simple biomaterial of natural origin, like silk, is also bioactive, the creation of a conduit equipped with a multi-layered wall can be a simple but effective response in a repair intervention of nerve damage with loss of substance (Alessandrino et al., 2019b).

SilkBridge^TM^ conduit is a tubular device whose wall is made by silk fibroin, a natural polymer produced by the silkworm *Bombyx mori*, that displays an optimum combination of strength and elasticity to withstand clinical operation stresses, such as manipulation and suturing during implantation, to resist deformation caused by biomechanical *in vivo* stresses, and to avoid channel collapse since compression can result in damage to the growing axon (Altman et al., 2003; Rockwood et al., 2011; Catto et al., 2015; Thurber et al., 2015). The wall of SilkBridge^TM^ is composed by three layers manufactured using a new combination of electrospinning and textile technologies that allows combination of micro- (to optimize the mechanical properties) and sub micro- (to maximize the biological characteristics of the material) fibrous elements (Alessandrino et al., 2019a; Alessandrino et al., 2019b).

In a previous work (Alessandrino et al., 2019b), the mechanical, structural and biological proprieties of SilkBridge^TM^ have been demonstrated. The wall thickness of about 0.5 mm and the wall porosity > 80% fall in the optimum range of geometric parameters able to ensure nutrient, oxygen, and metabolite transport and exchange with the surrounding environment, thus providing support to the regenerating axon (Kokai et al., 2009; Nectow et al., 2012; Chiono and Tonda-Turo, 2015). The conduit presents an optimal capability to resist to compression stresses, being able to resist to both physiological and pathological compressive stresses (Topp and Boyd, 2006), thus meeting an indispensable property for clinical application. From the biological point of view, the conduit has proven to be a good substrate for Schwann cells growth and proliferation, as well as for the differentiation and axonal elongation of neurons *in vitro*. Moreover, 2 weeks after implantation *in vivo* the conduit demonstrated a good integration with the surrounding tissues, absence of inflammation and scar formation.

In the current study we confirmed the optimal mechanical and biological properties, as well as the *in vivo* biocompatibility of the novel SilkBridge^TM^ conduit, which showed its great potential to sustain peripheral nerve regeneration at mid (4 weeks) and longer (12 and 24 weeks) time points in a model of rat median nerve injury. Indeed, histological analysis at 4 weeks showed the progression of nerve regeneration process alongside the conduit, demonstrated by the presence of many myelinated fibers in the proximal and mid portion of the conduit, and the approaching at the distal portion, and the good integration with the surrounding tissue, demonstrated by the whole colonization of both the lumen and the wall of the conduit by extracellular matrix and different cell types, the presence of a thin layer of connective tissue surrounding the outer side of the conduit, the formation of many blood vessels and the absence of any foreign body reaction.

At longer time points (12 and 24 weeks), the SilkBridge^TM^ conduit led to a very good functional and morphological recovery of the median nerve, similar to that observed with the reference autograft nerve reconstruction. The functional recovery, assayed by means of the grasping test and muscle weight, showed a stably motor performance of the finger flexor muscles, with no statistically nor clinically relevant difference in the SilkBridge^TM^ conduit group and in the Autograft group. The delay of functional recovery of the experimental group with respect to the control autograft group can be justified by the different repair technique and it is in line with the reconstruction through different conduits (Geuna et al., 2016). Morphological and morphometric analyses reflected the good performance of functional tests. Indeed, after 24 weeks, nerve fibers size, maturation, and organization of experimental and control groups levelled, suggesting a positive outcome of the regeneration process driven by the SilkBridge**^TM^** conduit. To be noticed, in the case of axon diameter and fiber diameter outcomes the statistically significant differences between the two groups that were detected refers to dimensions less than a micrometer, with no reliable confidence intervals, that could be considered clinically irrelevant.

Finally, special attention was paid to analyze the behavior of the silk material once implanted *in vivo*, with a particular regard to integration with regenerating tissues and to degradation over time. In the time points investigated, a large number of newly formed blood vessels were found in the SilkBridge^TM^ conduit during nerve regeneration and these vessels were mainly located at the periphery of the regenerated tissue, very close to the multi-layered wall and also between the three layers of the wall. The absence of this situation in the control group, at both experimental times, suggests that SilkBridge^TM^ through formation of blood vessels may have created a favorable oxygen-glucose environment in the nerve conduit, being therefore partly responsible for such a positive pro-regenerative effect (Wang et al., 2018; Chouhan and Mandal, 2020). A very important feature of tissue design is the rate of the scaffold degradation, the importance of the balance between the decomposing time of the biomaterial and the rate of tissue regeneration. Moreover, in case of silk fibroin, the velocity and the extent of degradation may be strictly connected with the structural characteristics of the polymers, the biological site of implantation, and the presence of different sources of mechanical and chemical stresses (Taddei et al., 2006). In this context and given the complex structure of SilkBridge^TM^ with its multilayer wall, a careful high-resolution optical analysis and a quantification through ultrastructure images have allowed to appreciate the first degradation phenomena, which in SilkBridge^TM^ have been observed at the level of ES-o and ES-i wall layers. The layers of the conduit wall, consisting of silk electrospun fibers, were in fact firstly intercalated with new cellular elements and extracellular matrix, thus losing their original compactness and subsequently, at long times after implantation, their electrospun fibers significantly reduced in the diameter.

Altogether, the present results and the previous ones (Alessandrino et al., 2019b) represent an important achievement for the implementation of clinical studies aimed at investigating the safety and efficacy of the newly designed SF-based nerve conduit, which is intended as an “off-the-shelf” device to be used as it is, without the need of adding neurotrophic and/or angiogenic factors or cells. These very encouraging results allowed us to proceed quickly towards the submission of a first-in-human clinical study aimed at evaluating the reconstruction of digital nerve defects in humans using SilkBridge**^TM^** nerve conduit (ClinicalTrials.gov identifier: NCT03673449). The study has already started at the Department of Plastic Surgery and Hand Surgery of the University Hospital of Zurich. Four out of 15 patients have been enrolled and implanted with SilkBridge**^TM^** nerve conduit to repair a digital nerve gap.

## Data Availability Statement

The datasets generated for this study are available on request to the corresponding author.

## Ethics Statement

The animal study was reviewed and approved by the Ethic Experimental Committee of the University of Turin (Ministry of Health project number 864/2016).

## Author Contributions

FF supported the surgery, carried out analysis, contributed to designed experiments and the data analysis, wrote, and revised the manuscript. LM performed *in vivo* functional experiments, carried out the morphological and stereological analysis, wrote, and revised the manuscript. AC carried out surgery on animals. GR performed surgery, carried out stereological and morphological analysis, wrote and revised the manuscript, and contributed to designed experiments. SG supported the study, designed experiments, and contributed to the data analysis. AA carried out the manufacturing and the mechanical characterization of SilkBridge^TM^, revised the manuscript, and contributed to design the experiments. GF and GB carried out the manufacturing and the mechanical characterization of SilkBridge^TM^, contributed to designed experiments, and provided guidance throughout the entire study. MB, VV, and PP supported the manufacturing and the mechanical characterization of SilkBridge^TM^ conduit. All authors contributed to the article and approved the submitted version.

## Conflict of Interest

This study is sponsored by Silk Biomaterials srl. GF is stock owners and consultant of the sponsoring organization. AA is stock owners and employee of the sponsoring organization. GB, MB, and VV are employees of the sponsoring organization. FF, LM, GC, GR, and SG are consultants of the sponsoring organization. The remaining authors declare that the research was conducted in the absence of any commercial or financial relationships that could be construed as a potential conflict of interest.

## Supplementary Material

FIGURE S1 Equivalence test on functional analysis. **(A)** Grasping test: The equivalence test was statistically significant (*t*[169.44] = −29.660, *p* < 0.000) and the null-hypothesis test was statistically significant (*t*[169.44] = 2.881, *p* < 0.004) revealing that the observed effect of Autograft superior to SilkBridge^TM^ of about 80.2 gr (*p* = 0, 95% CI: 63.99–96.42), can be considered overlapped. **(B)** Finger flexor superficialis muscles weight: The equivalence test was not statistically significant (*t*[27] = 5.773, *p* < 0.000) and the null-hypothesis test was statistically not significant (*t*[27] = −0.734, *p* = 0.469) revealing that the observe higher weight in Autograft group 0.003 gr is not statistically different (*p* = 0.483, 95% CI: −0.00–0.01).

FIGURE S2 Equivalence test on morphoquantitative evaluation of nerve regeneration. **(A)** Cross-sectional area: The equivalence test (*t*[12,53] = 1.489, *p* = 0.919) and the null-hypothesis test (*t*[27] = −0.734, *p* = 0.469) revealed that the Autograft had a higher cross-sectional area of about 0.049 mm^2^ (*p* < 0.007, 95% CI: 0.01–0.08). **(B)** Density of myelinated fibers: The equivalence test (*t*[18,87] = 0.323, *p* = 0.375) and the null-hypothesis test (*t*[18,87] = −0.990, *p* = 0.335) revealed an higher density of myelinated fibers in the SilkBridge^TM^ conduit group of about 2293 fibers/mm^2^; not statistically relevant (*p* = 0.302, 95% CI: −2208.85–6796.35). **(C)** Total number of myelinated fibers: the equivalence test and the null-hypothesis test were not a significant (*t*[22,43] = 0.963, *p* = 0.827; *t*[22,43] = 1.982, *p* = 0.061). **(D)** g-Ratio: the equivalence test and the null-hypothesis test were not a significant (*t*[18,71] = −1.173, *p* = 0.128; *t*[18,71] = 1.347, *p* = 0.194). **(E)** Axons diameter: the equivalence test and the null-hypothesis test were not a significant (*t*[14,7] = −0.045, *p* = 0.483; *t*[14,7] = 1.811, *p* = 0.0906). **(F)** Fiber diameter: the equivalence test and the null-hypothesis test were not a significant (*t*[23,28] = −0.233, *p* = 0.591; *t*[23,28] = −1.937, *p* = 0.0645). **(G)** Myelin thickness: the equivalence test and the null-hypothesis test were not a significant (*t*[20,85] = −0.262, *p* = 0.398; *t*[20,85] = 0.949, *p* = 0.353).

TABLE S1 Mechanical properties of SilkBridge^TM^ conduit.
