# Supplementary figures and images for: Preclinical Validation of SilkBridgeTM for Peripheral Nerve Regeneration
Source: Front Bioeng Biotechnol. 2020 Aug 7;8:835. doi: 10.3389/fbioe.2020.00835 (PMC7426473; doi:10.3389/fbioe.2020.00835)

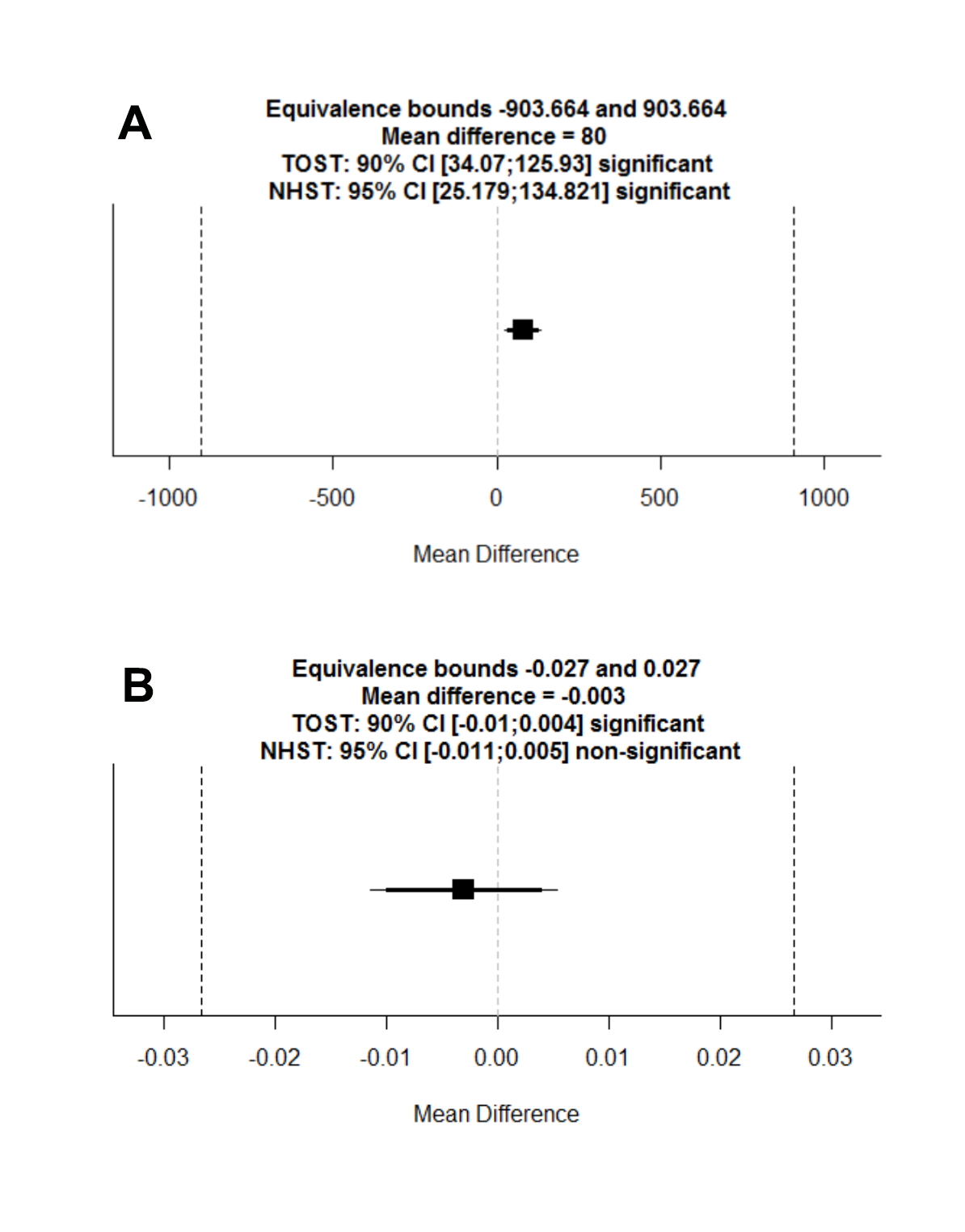

Supplement: FIGURE S1 — Equivalence test on functional analysis. (A) Grasping test: The equivalence test was statistically significant (t[169.44] = −29.660, p < 0.000) and the null-hypothesis test was statistically significant (t[169.44] = 2.881, p < 0.004) revealing that the observed effect of Autograft superior to SilkBridgeTM of about 80.2 gr (p = 0, 95% CI: 63.99–96.42), can be considered overlapped. (B) Finger flexor superficialis muscles weight: The equivalence test was not statistically significant (t[27] = 5.773, p < 0.000) and the null-hypothesis test was statistically not significant (t[27] = −0.734, p = 0.469) revealing that the observe higher weight in Autograft group 0.003 gr is not statistically different (p = 0.483, 95% CI: −0.00–0.01). [file Image_1.TIF]

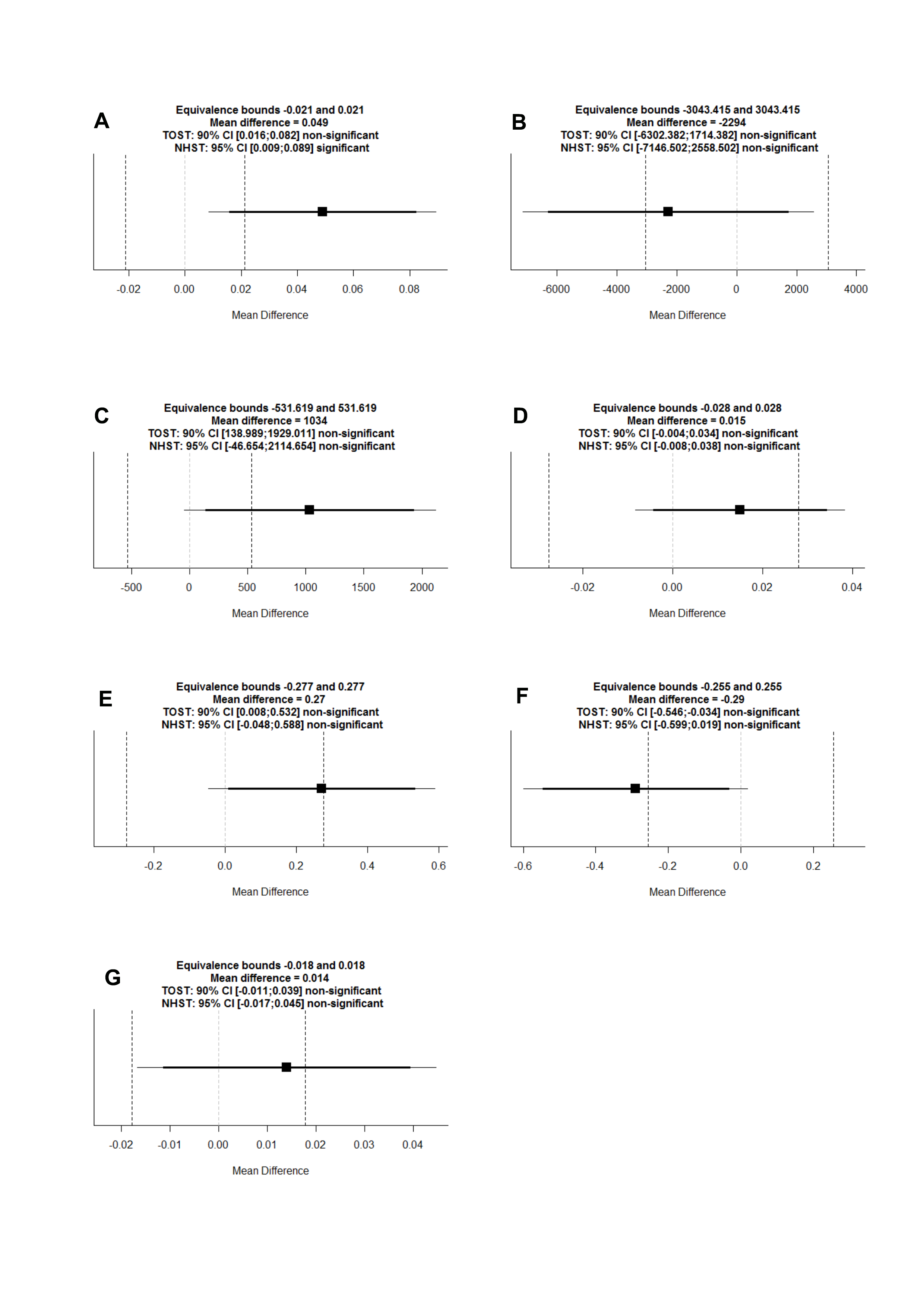

Supplement: FIGURE S2 — Equivalence test on morphoquantitative evaluation of nerve regeneration. (A) Cross-sectional area: The equivalence test (t[12,53] = 1.489, p = 0.919) and the null-hypothesis test (t[27] = −0.734, p = 0.469) revealed that the Autograft had a higher cross-sectional area of about 0.049 mm2 (p < 0.007, 95% CI: 0.01–0.08). (B) Density of myelinated fibers: The equivalence test (t[18,87] = 0.323, p = 0.375) and the null-hypothesis test (t[18,87] = −0.990, p = 0.335) revealed an higher density of myelinated fibers in the SilkBridgeTM conduit group of about 2293 fibers/mm2; not statistically relevant (p = 0.302, 95% CI: −2208.85–6796.35). (C) Total number of myelinated fibers: the equivalence test and the null-hypothesis test were not a significant (t[22,43] = 0.963, p = 0.827; t[22,43] = 1.982, p = 0.061). (D) g-Ratio: the equivalence test and the null-hypothesis test were not a significant (t[18,71] = −1.173, p = 0.128; t[18,71] = 1.347, p = 0.194). (E) Axons diameter: the equivalence test and the null-hypothesis test were not a significant (t[14,7] = −0.045, p = 0.483; t[14,7] = 1.811, p = 0.0906). (F) Fiber diameter: the equivalence test and the null-hypothesis test were not a significant (t[23,28] = −0.233, p = 0.591; t[23,28] = −1.937, p = 0.0645). (G) Myelin thickness: the equivalence test and the null-hypothesis test were not a significant (t[20,85] = −0.262, p = 0.398; t[20,85] = 0.949, p = 0.353). [file Image_2.TIF]
