# Supplementary material for: Preclinical Validation of SilkBridgeTM for Peripheral Nerve Regeneration
Source: Front Bioeng Biotechnol. 2020 Aug 7;8:835. doi: 10.3389/fbioe.2020.00835 (PMC7426473; doi:10.3389/fbioe.2020.00835)
Supplement: TABLE S1 — Mechanical properties of SilkBridgeTM conduit. [file Table_1.DOCX]

| Tensile properties | | | | | Suture retention strength | |
| --- | --- | --- | --- | --- | --- | --- |
| Load (N) | Strain (%) | Stress (MPa) | Modulus Phase 1 (MPa) | Modulus Phase 2 (MPa) | Load (gf)  9/0 Suture | Load (gf)  5/0 Suture |
| 26.7 ± 2.3 | 75.9 ± 6.3 | 8.1 ± 0.7 | 3.3 ± 0.6 | 18.2 ± 0.7 | > 117 | 461 ± 141 |

**TABLE 1**
